# Generation of three induced pluripotent stem cell lines from hypertrophic cardiomyopathy patients carrying *TNNI3* mutations

**DOI:** 10.1016/j.scr.2021.102597

**Published:** 2021-11-12

**Authors:** Shane Rui Zhao, Mengcheng Shen, Chelsea Lee, Yanjun Zha, Julio V. Guevara, Matthew T. Wheeler, Joseph C. Wu

**Affiliations:** a Stanford Cardiovascular Institute, Stanford University School of Medicine, Stanford, CA, USA; b Division of Cardiovascular Medicine, Department of Medicine, Stanford University School of Medicine, Stanford, CA, USA

## Abstract

Hypertrophic cardiomyopathy (HCM) is a common inherited heart disease with a prevalence of about 0.2%. HCM is typically caused by mutations in genes encoding sarcomere or sarcomere-associated proteins. Here, we characterized induced pluripotent stem cell (iPSC) lines generated from the peripheral blood mononuclear cells of three HCM patients each carrying c.433C > T, c.610C > T, or c.235C > T mutation in the *TNNI3* gene by non-integrated Sendai virus. All of the three lines exhibited normal morphology, expression of pluripotent markers, stable karyotype, and the potential of trilineage differentiation. The cardiomyocytes differentiated from these iPSC lines can serve as useful tools to model HCM *in vitro*.

## 1. Resource table

**Table:** 

Unique stem cell lines identifier	1) SCVIi017-A 2) SCVIi018-A 3) SCVIi019-A

Alternative name(s) of stem cell lines	
Institution	Stanford Cardiovascular Institute, Stanford, CA, US
Contact information of distributor	Joseph C. Wu, joewu@stanford.edu
Type of cell lines	iPSC
Origin	Human
Additional origin info required *for human ESC or iPSC*	Age: 43 (SCVIi017-A), 23 (SCVIi018-A), 23 (SCVIi019-A) Sex: Female (SCVIi017-A), Male (SCVIi018-A), Male (SCVIi019-A) Ethnicity if known: Not Hispanic or Latino (all three lines)
Cell Source	Blood
Clonality	Clonal
Associated disease	Hypertrophic cardiomyopathy (HCM)
Gene/locus	*TNNI3* c.433C > T (SCVIi017-A) *TNNI3* c.610C > T (SCVIi018-A) *TNNI3* c.235C > T (SCVIi019-A)
Date archived/stock date	Aug 3rd, 2021
Cell line repository/bank	https://hpscreg.eu/cell-line/SCVIi017-A https://hpscreg.eu/cell-line/SCVIi018-A https://hpscreg.eu/cell-line/SCVIi019-A
Ethical approval	The generation of the lines was approved by the Administrative Panel on Human Subjects Research (IRB) under **IRB #29904** “Derivation of Human Induced Pluripotent Stem Cells (Biorepository)”.

## 2. Resource utility

Three induced pluripotent stem cell (iPSC) lines were generated from three hypertrophic cardiomyopathy (HCM) patients each carrying different heterozygous mutation in the *TNNI3* gene. These fully characterized iPSC lines can be differentiated into cardiomyocytes to understand the complex pathogenic mechanisms of HCM.

## 3. Resource details

HCM is a genetic disorder characterized by left ventricular hypertrophy. HCM is predominantly caused by mutations in genes encoding sarcomere or sarcomere-associated proteins (Lan et al., 2013; Marian & Braunwald, 2017; Wu et al., 2019). Thin filaments of the sarcomeres are composed of tropomyosin, troponin and actin (van der Velden & Stienen, 2019). The *TNNI3* gene encodes cardiac troponin I (cTnI), a subunit of the troponin complex. Notably, mutations in *TNNI3* have been reported in 2%–7% of HCM cases (Mogensen et al., 2004).

**Fig. 1.**
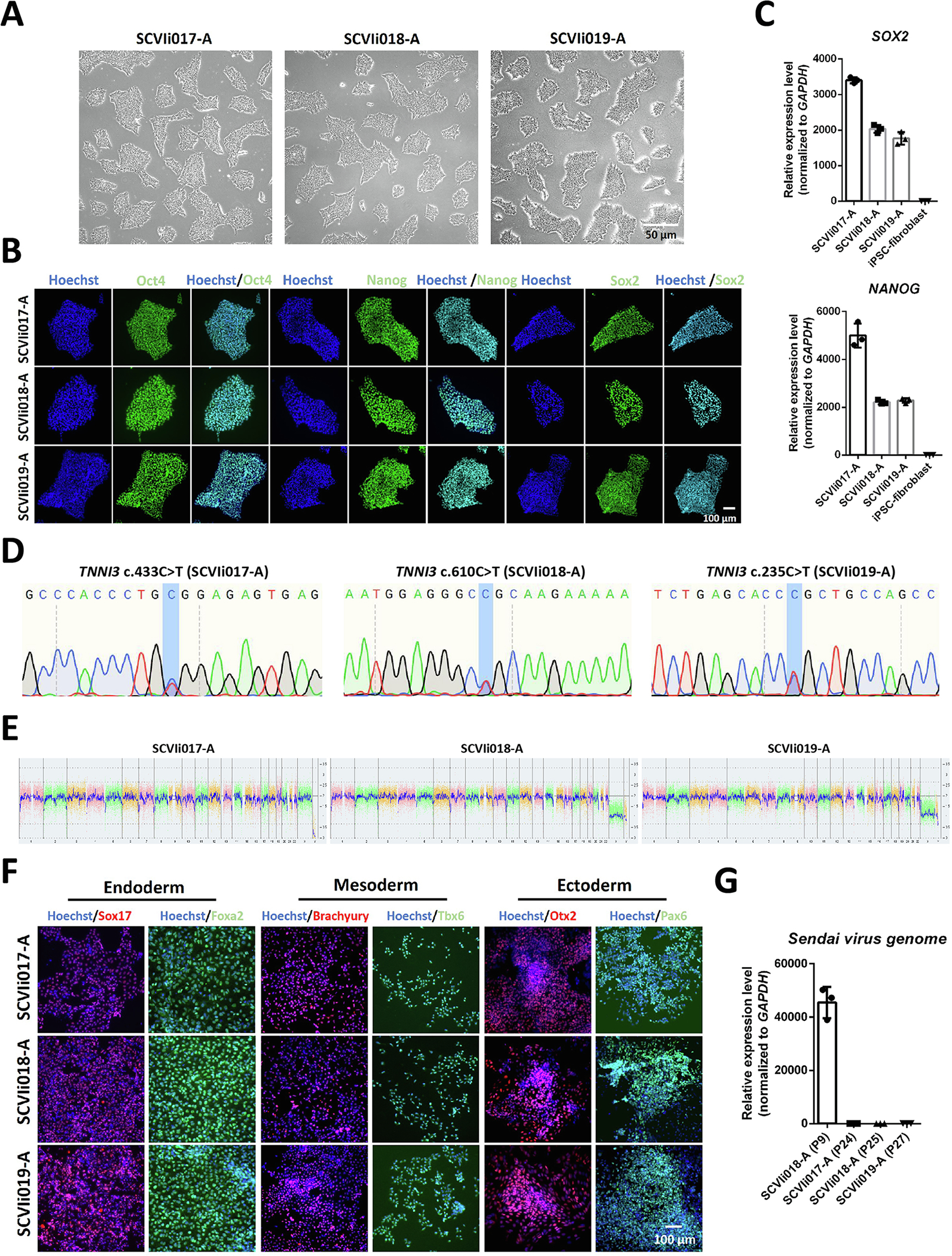
Characterization of iPSC lines derived from hypertrophic cardiomyopathy patients carrying *TNNI3* mutations. (A) Brightfield images of the iPSC lines. Scale bar, 50 μm. (B) Immunofluorescent staining images for pluripotency markers OCT4, SOX2, and NANOG. Scale bar, 100 μm. (C) Quantification of *NANOG* and *SOX2* expression by RT-qPCR. IPSC-derived fibroblasts were used as a negative control. (D) Results of Sanger sequencing showing *TNNI3* mutations. (E) Results of KaryoStat assay. (F) Immunofluorescent staining images for markers of three germ layers. Scale bar, 100 μm. (G) Quantification of Sendai virus (SEV) expression by RT-qPCR.

In this report, we generated three iPSC lines SCVIi017-A, SCVIi018-A, and SCVIi019-A from three HCM patients each carrying distinct mutation in *TNNI3*. Peripheral blood mononuclear cells (PBMCs) collected from these patients were reprogrammed into iPSCs using Sendai virus carrying reprogramming factors *OCT4*, *SOX2*, *KLF4*, and *c-MYC*. All of the three iPSC lines showed typical iPSC morphology (Fig. 1A). High expression levels of pluripotency markers were confirmed by immunofluorescence staining and reverse transcription quantitative polymerase chain reaction (RT-qPCR) (Fig. 1B and 1C). Genetic testing confirmed c.433C > T, c.610C > T and c.235C > T mutations in *TNNI3* of SCVIi017-A, SCVIi018-A, and SCVIi019-A, respectively (Fig. 1D). Neither reprogramming nor long-term maintenance compromised the karyotype integrity of these iPSC lines (Fig. 1E). All of the three iPSC lines demonstrated full potential to generate three lineages by expressing endoderm (Sox17 and Foxa2), mesoderm (Brachyury and Tbx6), and ectoderm (Otx2 and Pax6) markers (Fig. 1F). While trace amount of Sendai virus was detectable at early passages of iPSCs, it was absent at passages 24–27 (Fig. 1G). All iPSC clones were tested negative for mycoplasma (Supplementary Fig. 1). A set of 16 polymorphic short tandem repeats (STR) analysis confirmed the identicalness of the three iPSC lines to the patients’ PBMCs (data archived) (Table 1).

## 4. Materials and methods

### 4.1. Reprogramming

PBMCs were isolated and collected by gradient centrifugation from the peripheral blood of patients. PBMCs were isolated by Percoll separation (GE Healthcare) and purified by washing with DPBS buffer (Thermo Fisher Scientific). After replating, PBMCs were cultured in PBMC medium containing complete StemPro-34 medium (Thermo Fisher Scientific) supplemented with 100 ng/mL SCF (Peprotech), 100 ng/mL FLT3 (Thermo Fisher Scientific), 20 ng/mL IL-3 (Peprotech), 20 ng/mL IL-6 (Thermo Fisher Scientific), and 20 ng/mL EPO (Thermo Fisher Scientific). PBMCs were reprogrammed to iPSCs by the CytoTune®-iPSC Sendai Reprogramming Kit (Thermo Fisher Scientific) according to the manufacturer’s instructions. Briefly, transduced PBMCs were resuspended and plated. The StemPro™−34 medium was refreshed every two days. At day 7, the medium was changed to fresh StemMACS™ iPS-Brew XF medium (Miltenyi Biotechnology). Medium was refreshed every other day until day 10–15 post-infection when colonies were ready to be picked. Picked colonies were further expanded and frozen down for downstream applications.

### 4.2. Cell culture

iPSCs were cultured in StemMACS™ iPS-Brew XF medium in 6-well plates coated with Matrigel (Corning) at a dilution of 1:400 in a humidified incubator at 37 °C with 5% CO_2_. Medium was changed every other day. iPSCs were passaged at a ratio of 1:6 to 1:12. Y-27632 (10 μM), a potent inhibitor of ROCK1 (Selleck Chemicals), was added in the medium during the first 24 h of cell replating to improve cell survival and attachment.

### 4.3. Immunofluorescence staining

iPSCs at passages 15–20 and iPSC derivatives were fixed with 4% paraformaldehyde for 15 min at room temperature (RT), permeabilized with 0.3% Triton X-100 (Sigma) for 10 min at RT, and blocked with 3% bovine serum albumin (BSA, Sigma) for 30 min at RT. Then cells were incubated with primary antibodies overnight at 4 °C and fluorescence-conjugated secondary antibodies for 60 min at RT. Cell nuclei were counter stained with Hoechst 33342 (Thermo Fisher Scientific) for 5 min at RT. Images were captured using an inverted fluorescence microscope. The antibody information and dilution ratios are listed in Table 2.

### 4.4. Trilineage differentiation potential assay

iPSCs at passages 15–20 were differentiated using the STEMdiff™ trilineage differentiation kit (Stemcell Technologies) according to the manufacturer’s instructions. Differentiations were assessed by the expressions of classical lineage markers in each germ layer.

### 4.5. RT-qPCR

Total RNA was extracted by miRNeasy Micro Kit (Qiagen). RT-qPCR was performed by iScript™ Reverse Transcription Supermix (Bio-rad) according to the manufacturer’s instructions. iPSCs at passages 15–20 were used for the detection of pluripotency markers. iPSCs at passages 24–27, as well as early passage (P9), were used for the detection of Sendai virus genome.

### 4.6. Karyotyping

A total of 2 × 10^6^ iPSCs were collected from each line between passages 11–15 and analyzed using the KaryoStat™ assay (Thermo Fisher Scientific).

### 4.7. Short tandem repeat (STR) analysis

Genomic DNAs of PBMCs and iPSCs at passages 15–20 were isolated by QuickExtract™ DNA Extraction Solution (Lucigen). STR analysis was performed using a CLA IdentiFiler™ Direct PCR Amplification Kit (Thermo Fisher Scientific). Capillary electrophoresis was performed on ABI3130xl by the Stanford Protein Nucleic Acid (PAN) Facility.

### 4.8. Mycoplasma detection

Mycoplasma detection was performed by a MycoAlert™ Detection Kit (Lonza) according to the manufacturer’s instructions.

### 4.9. DNA sequencing

Genomic DNA was isolated from iPSCs at passages 15–20 using the QuickExtract™ DNA Extraction Solution (Lucigen) and amplified by PCR. Information of the designed primers was listed in Table 2. Purified PCR products were subjected to Sanger sequencing. The presence of *TNNI3* mutations was identified by aligning the Sanger sequencing data with wildtype *TNNI3* sequence using SnapGene software.

## Supplementary Material

1

## Figures and Tables

**Table 1:** Characterization and validation.

Classification	Test	Result	Data

Morphology	Photography brightfield	Visual record of the line: normal	Fig. 1 panel A
Phenotype	Qualitative analysis Immunocytochemistry	Positive expression of pluripotency markers: Oct3/4, Nanog, Sox2	Fig. 1 panel B
	Quantitative analysisRT-qPCR	*NANOG* and *SOX2* are highly expressed	Fig. 1 panel C
Genotype	Whole genome array(KaryoStat™ Assay)Resolution 1–2 Mb	Normal karyotype: 46, XY and 46, XX	Fig. 1 panel E
Identity	Microsatellite PCR (mPCR) or STR analysis	N/A 16 loci tested, all matched	N/A Submitted in archive with journal
Mutation analysis (IF APPLICABLE)	Sequencing	Heterozygous Heterozygous Heterozygous	Fig. 1 panel D
	Southern blot or WGS	N/A	N/A
Microbiology and virology	Mycoplasma	Mycoplasma testing by luminescence. Negative	Supplementary Fig. 1
Differentiation potential	Directed differentiation	Positive expression of three germ layer markers by immunocytochemistry	Fig. 1 panel F
List of recommended germ layer markers	Expression of these markers has to be demonstrated at mRNA (RT-PCR) or protein (IF) levels, at least 2 markers need to be shown per germ layer	Ectoderm: Pax6, Otx2 Endoderm: Sox17, Foxa2 Mesoderm: Brachyury, Tbx6	Fig. 1 panel F
Donor screening (OPTIONAL)	HIV 1 + 2 Hepatitis B, Hepatitis C	N/A	N/A
Genotype additional info (OPTIONAL)	Blood group genotyping HLA tissue typing	N/A N/A	N/A N/A

**Table 2:** Reagents details.

Antibodies used for immunocytochemistry/flow-cytometry				

	Antibody	Dilution	Company Cat #	RRID

Pluripotency Markers	Rabbit Anti-Nanog	1:200	ProteintechCat# 142951-1-AP	RRID: AB_1607719
Pluripotency Markers	Mouse IgG2bκ Anti-Oct-3/4	1:200	Santa CruzBiotechnologyCat# sc-5279	RRID: AB_628051
Pluripotency Markers	Mouse IgG1κ Anti-Sox2	1:200	Santa CruzBiotechnologyCat# sc-365823	RRID: AB_10842165
Ectoderm marker	Goat Anti-Otx2	1:200	R&D SystemsCat# 963273	RRID: AB_2157172
Ectoderm marker	Rabbit Anti-Pax6	1:100	Thermo FisherScientificCat# 42-6600	RRID: AB_2533534
Endoderm marker	Goat Anti-Sox17	1:200	R&D SystemsCat# 963121	RRID: AB_355060
Endoderm marker	Rabbit Anti-Foxa2	1:250	Thermo FisherScientificCat# 701698	RRID: AB_2576439
Mesoderm marker	Goat Anti-Brachyury	1:200	R&D SystemsCat# 963427	RRID: AB_2200235
Mesoderm marker	Rabbit Anti-Tbx6	1:200	Thermo FisherScientificCat# PA5-35102	RRID: AB_2552412
Secondary antibody	Alexa Fluor 488 Goat Anti-Mouse (H + L)	1:500	Thermo FisherScientificCat# A-32723	RRID: AB_2633275
Secondary antibody	Alexa Fluor 488 Goat Anti-Rabbit (H + L)	1:500	Thermo FisherScientificCat# A-32731	RRID: AB_2633280
Secondary antibody	Alexa Fluor 594 Donkey Anti-Goat (H + L)	1:500	Thermo FisherScientificCat# A-11058	RRID: AB_2534105
	Primers			

	Target	Size of band	Forward/Reverse primer (5′-3′)	
Sendai virus plasmids (qPCR)	Sendai virus genome	181 bp	Mr04269880_mr	
Pluripotency marker (qPCR)	*SOX2*	258 bp	Hs04234836_s1	
Pluripotency marker (qPCR)	*NANOG*	327 bp	Hs02387400_g1	
House-keeping gene (qPCR)	*GAPDH*	91 bp	Hs02758991_g1	
Genotyping	TNNI3 c.433C > THeterozygous	525 bp	Forward: CCATGGGTTGGGAAACAGAAAATReverse: GCCTTAGCCCACACTCACCTTCT	
Genotyping	TNNI3 c.610C > THeterozygous	593 bp	Forward: GGAGGGAAGACAGGGATTCTTGAReverse: GTGTGTCCATGTGTCCACCTGTC	
Genotyping	TNNI3 c.235C > THeterozygous	582 bp	Forward: ATCCTTCCTTGCTCCATCTCACCReverse: TGGGTAAGGACAGCCATATTGGA	

RRID Requirement for antibodies: use http://antibodyregistry.org/ to retrieve RRID for antibodies and include ID in table as shown in examples.
